# Investigating the role of cortisol and growth hormone in fatty liver development: fatty liver index in patients with pituitary adenomas

**DOI:** 10.1007/s11102-016-0726-1

**Published:** 2016-05-18

**Authors:** Matthias K. Auer, Günter K. Stalla, Mareike R. Stieg

**Affiliations:** RG Neuroendocrinology, Max Planck Institute of Psychiatry, Kraepelinstrasse 2-10, 80804 Munich, Germany

**Keywords:** Adrenal insufficiency, Cushing’s disease, Growth hormone, Fatty liver, Cortisol

## Abstract

**Purpose:**

Non-alcoholic fatty liver disease (NAFLD) is a hallmark of the metabolic syndrome and has been shown to be an independent predictor of cardiovascular mortality. Although glucocorticoids and growth hormone are known to be implicated in its pathophysiology, it has only rarely been investigated in the context of patients with pituitary insufficiency or former cortisol excess.

**Methods:**

Case–control study in patients with biochemically controlled Cushing’s disease (CD; N = 33) and non-functioning pituitary adenomas (NFPA; N = 79). NAFLD was estimated by calculating the fatty liver index (FLI) including BMI, waist circumference, GGT and triglyceride levels.

**Results:**

Although there was no difference in FLI between patients with NFPA and CD, we identified average daily hydrocortisone (HC) intake in those with adrenal insufficiency to be an independent predictor of FLI (β = 1.124; *p* = 0.017), even after adjusting for BMI and waist circumference. In line, those with a FLI > 60 were also taking in average significantly more HC per day than those with a score <60 (21.05 mg ± 5.9 vs. 17.9 mg ± 4.4; *p* = 0.01). FLI was also the best independent predictor for HbA1c and fasting glucose levels (both *p* = 0.001). Growth hormone deficiency and replacement therapy were not associated with FLI in either group.

**Conclusions:**

While HC dosage affects FLI as an estimate of NFLD in patients with CD and NFPA, the benefit of GH replacement still needs to be determined. In contrast to reports in CD patients with active disease, NAFLD in those with biochemical control was not different from NFPA patients.

## Introduction

Non-alcoholic fatty liver disease (NAFLD) is the most common liver disease in western populations and it is closely linked to the development of the metabolic syndrome [1]. The term encompasses simple steatosis of the liver as well as non-alcoholic steatohepatitis (NASH). NASH in turn can ultimately result in liver fibrosis and cirrhosis and increases also the risk for hepatocellular carcinoma [2]. Beyond its contribution to metabolic disturbances, it is also an independent predictor of cardiovascular mortality [3].

Patients with pituitary adenomas in general and those with Cushing’s disease (CD) in particular have an increased cardiovascular risk. In case of CD this is even true for those who have achieved biochemical control [4]. But also patients with non-functioning pituitary adenomas (NFPA), in particular those with impairment of pituitary function, have an increased risk to die from cardiovascular diseases [5]. In particular growth hormone deficiency and adrenal insufficiency may explain for the increase standard mortality ratio in these patients [6]. Pituitary insufficiencies are also common in CD patients [7], therefore potentially contributing in addition to the detrimental effects of long-lasting glucocorticoid (CG) excess to metabolic disturbances and cardiovascular mortality [8, 9]. The role of pituitary insufficiency on cardiovascular parameters in Cushing’s disease is only poorly understood and provided controversial results so far [8–11].

NAFLD has only rarely been investigated in the context of pituitary adenomas [12–14], though it represents and interesting candidate to explain for metabolic disturbances and cardiovascular mortality. It is of special interest in this particular patient population as the involvement of GC as well as growth hormone (GH) is suggested to play a fundamental role in the development of NAFLD [15, 16].

In line, in a study with CD patients with active disease, the estimated prevalence of NAFLD by CT measurements was 20 % and closely related to visceral adiposity [12]. Vice versa, in the general population, increased exposure to GCs is associated with NAFLD [16]. These findings underline the critical role of hepatic *e*xposure to GCs for the development of NAFLD.

The role of the GH/insulin-like growth factor-1(IGF-1) system in this context is less clear. Both GH and IGF-1 are however believed to be important in the regulation of hepatic lipid metabolism [17]. Growth hormone deficiency (GHD) appears to be associated with increased hepatic lipid content [14], however interventional studies have yielded controversial results [13, 14].

Although currently the diagnosis of NAFLD requires a liver biopsy, non-invasive procedures have been developed in the past that show adequate concordance with histological results. This includes the fatty-liver-index (FLI) [18] which has been demonstrated to be a useful tool to predict the presence of NAFLD as it shows high accordance with imaging [19] as well as histological criteria for NAFLD [20]. The index has been shown to be a useful predictor of arteriosclerosis development and all-cause mortality, independent of established classical risk factors in particular in those patients with a score ≥60 [21].

In the present study we therefore investigated the effects of long-lasting former cortisol excess in CD on FLI as a marker of NAFLD in comparison to patients with NFPA, to disentangle the different effects of past cortisol excess, present adrenal insufficiency and GHD on NAFLD. We also investigated if FLI would be a good predictor in this special population for disturbances in glucose metabolism and further classical cardiometabolic risk factors.

## Methods

Suitable patients were selected from a patient cohort of the department of Clinical Neuroendocrinology of the Max Planck Institute of Psychiatry in Munich, Germany who had been included in the Network of Excellence for Neuroendocrine Tumors Munich (NeoExNET), a registry and biobank alliance of several regional academic institutions. Data necessary for the calculation of the FLI had not been systematically assessed before 2012, so only patients that had visited our department between the years 2012–2014 were included in the present analysis. We only included patients with CD who were biochemically cured for at least 12 month prior to the analysis (N = 33). Five CD patients were receiving CD-specific medications at the time of study inclusion (one cabergolin, three azol-derivates and one pasireotide).

We further included patients with NFPA (N = 79). Patients were excluded if GHD had not been documented by appropriate stimulation tests [22]. We also only included patients who had been diagnosed with GHD for at least 1 year prior to the investigation. To compare for the sustained effects of GH-substitution, we also only included those in the GH-treated group who had at least been treated with GH for 12 months prior to the study inclusion.

Adrenal insufficiency had to be documented by adequate stimulation tests [23]. Evaluation of further pituitary function, respectively adequateness of treatment based on basal measurements of thyroid-stimulating hormone (TSH), free thyroxine, free triiodothyronine, luteinizing hormone (LH), follicle-stimulating hormone (FSH), and total testosterone (in men) or oestradiol (in women). Further parameters were assessed after an overnight fast between 8 and 11:30 a.m. using routine clinical methods at the local laboratory. IGF-1 and GH were measured on the IMMULITE 2000^®^ analyzer (Siemens, Los Angeles, CA).

In women in the fertile age range, secondary hypogonadism was defined as low LH/FSH in women with secondary amenorrhea for at least 1 year. In postmenopausal women, secondary hypogonadism was documented when gonadotropin levels were inappropriately low for the postmenopausal age. In men, secondary hypogonadism was defined as a low testosterone level with an inappropriate low LH/FSH and secondary hypothyroidism as low free T4 (FT4) with an inappropriately low serum TSH. Patients with adrenal insufficiency were treated with conventional hydrocortisone usually divided into 2–3 doses per day. Secondary hypothyroidism was treated with levothyroxine. LH/FSH deficiency was treated with testosterone in men (gel or intramuscular injections) and with estrogen/progesterone replacement in women or in case of past hysterectomy with estradiol only. Most women were receiving transdermal estradiol preparations.

Patients were also excluded if average alcohol intake was >30 g/day for men and >20 g for women or if they had known cirrhosis, hepatitis B or C. Height and weight were measured to the nearest 0.5 cm and 0.1 kg, respectively. Waist circumference was measured to the nearest 0.5 cm. History of medication intake and comorbidities was retrieved from the patient files and by interview. FLI was calculated according to an equation including BMI, waist circumference, GGT and triglyceride levels [18]. All patients gave written informed consent and the study was approved by the ethical review board of the Ludwig Maximilian University of Munich.

### Statistical analysis

Statistical analysis was conducted using SPSS 21.0 for Windows software (SPSS Inc). Quantitative data are expressed as mean ± standard deviation (SD) or standard error (SE), and categorical data are expressed as percentages. Differences between groups were performed using χ^2^-tests. Normal distribution of variables was tested by Kolmogorov–Smirnov-Test and skewed variables were log-transformed for further testing procedures. Differences between CD and NFPA patients were evaluated with an analysis of covariance (ANCOVA) controlling for age and gender which was different between the groups. Relations between the outcome variables and continuous variables were evaluated by univariate Pearson’s correlation coefficients or Spearman as appropriate. Multivariate linear regression models were used to investigate the relationship between depended and independent variables, including potential influential factors. Logistic regression analysis was used to test the independence of dichotomous outcome variables with their significant correlates in univariate models. Statistical tests were two-sided, and significance was set at a value of *p* < 0.05.

## Results

### General characteristics

In accordance with the general epidemiological characteristics of these two pituitary adenoma entities, significantly more patients with CD were women than patients with NFPA and they were also significantly younger (50.3 years ± 13.4 vs. 61.6 years ± 14.5; *p* = 0.037). Fewer CD patients had a macroadenoma at the time of diagnosis (*p* > 0.001) but radiotherapy was more frequently applied (*p* = 0.005). Adjusted mean systolic and diastolic blood pressure was not significantly different between NFPA and CD patients and same was true for those who had already been diagnosed and pretreated with antihypertensive drugs. Adjusted weight, BMI, and waist circumference did not differ between the groups. There was also no significant difference in the rate of patients with pre-diagnosed and pre-treated type 2-diabetes, intake of anti-hyperlipidemic drugs and also not for adjusted fasting glucose, HbA1c, total cholesterol, HDL, LDL, ALAT, ASAT, AP, GGT fasting triglyceride levels or IGF-1.

There was no significant difference in the total rate of pituitary insufficiency or total number of affected pituitary axes, but secondary hypogonadism (*p* = 0.007) and GHD (*p* = 0.015) was more common in NFPA patients than in those with CD. There was only a trend for more secondary adrenal insufficiencies in NFPA patients (*p* = 0.072) and as five patients had been bilaterally adrenalectomized in the course of CD, there was no significant difference with regard to the total rate of adrenal insufficiencies between the both groups. Average daily HC intake did not significantly differ between the groups. There was also no significant difference in overall untreated hypogonadism [defined as primary (=menopause) + secondary] and untreated GHD. FLI in NFPA patients in comparison to CD patients did not differ and same was true for the proportion of patient with an FLI above or below 60 (Table 1).

**Table 1:** General characteristics

	Cushing’s disease		NFPA		*p*
	N	%	N	%	
General characteristics					
Male	1	3	43	54.4	**<0.001**
Female	32	97	36	45.6	

	Mean	SD	Mean	SD	*p*
Age (years)	50.3	13.4	61.6	14.5	**0.04**

	N	%	N	%	*p*
Tumorsize					
Macroadenoma	9	27.3	78	98.7	**<0.001**
Microadenoma	23	69.7	1	1.3	
NA	1	3.0	0	0.0	
Treatment					
Surgery	33	100	72	91.1	0.712
Radiotherapy	13	39.4	12	15.2	**0.005**

	Mean	SE	Mean	SE	*p*
Anthropometry					
Weight (kg)	75	3.3	78	2.8	0.535
BMI (kg/m^2^)	26.6	1	27.8	0.6	0.317
Waist (cm)	94.2	3.1	94	2	0.952
Systolic blood pressure (mmHg)	128.8	3.1	136.9	1.8	0.370
Diastolic blood pressure (mmHg)	85.2	1.9	85.2	1.1	0.994

	N	%	N	%	*p*
Drug intake					
Anti-hypertensive treatment					
No	19	57.6	19	57.6	0.094
Yes	14	42.4	14	42.4	
Anti-hyperglycemic treatment					
No	29	87.9	68	86.1	0.798
Yes	4	12.1	11	13.9	
Dyslipidemia treatment					
No	28	84.8	59	74.7	0.239
Yes	5	15.2	20	25.3	

	Mean	SE	Mean	SE	*p*
Laboratory					
Fasting glucose (mg/dl)	87.3	2.8	84.1	1.7	0.367
Hba1c (%)	5.5	0.1	5.3	0	0.113
Total cholesterol (mg/dl)	212.3	8.2	216.4	5	0.690
HDL (mg/dl)	63.6	3.8	59.5	2.2	0.389
LDL (mg/dl)	136.7	7.2	140.1	4.3	0.708
Triglycerides (mg/dl)	145.3	15.9	141.9	10.1	0.866
Gamma-GT (U/l)	32.8	5.7	32.8	5.7	0.801
ALAT(U/l)	23.0	2.5	26.2	1.5	0.303
ASAT (U/l)	22.4	1.7	26.6	1	0.450
AP (U/l)	64.5	4.2	69.3	2.6	0.359

	N	%	N	%	*p*
FLI					
Mean FLI	47.6	6	49.1	3.6	0.846

	Mean	SE	Mean	SE	*p*
FLI < 60	23	69.7	26	32.9	0.208
FLI > 60	10	30.3	16	20.3	
IGF1 (ng/dl)	118.7	14.3	141	7.8	0.201
Pituitary function					
Pituitary insufficiency					
No	10	30.3	18	22.8	0.402
Yes	23	69.7	61	77.2	
Number of pituitary deficiencies					
0	8	24.2	15	19	**0.07**
1	9	27.3	11	13.9	
2	5	15.2	8	10.1	
3	7	21.2	18	22.8	
4	3	12.1	27	34.2	
Secondary hypogonadism					
No	20	60.6	26	32.9	**0.01**
Yes	13	39.4	53	67.1	
Menopause	6	18.2	9	11.4	0.724
Total sex steroid deficiency					
No	25	75.8	52	65.8	0.301
Yes	8	24.2	26	32.9	
Secondary adrenal insufficiency					
No	24	72.7	43	54.4	**0.07**
Yes	9	27.3	36	45.6	
Total adrenal insufficiency*					
No	19	57.6	43	54.4	0.072
Yes	14	42.4	36	45.6	

	Mean	SE	Mean	SE	*p*
Average daily hydrocortisone intake (mg; range 10–30 mg)	20.4	3.3	18.7	5.7	0.324

	N	%	N	%	*p*
Secondary hypothyroidism					
No	15	45.5	47	59.5	0.173
Yes	18	54.5	32	40.5	
GHD					
No	19	57.6	26	32.9	**0.02**
Yes	14	42.4	53	67.1	
Treated	7	21.2	29	36.7	0.109
Untreated	7	21.2	24	30.4	
Overt GHD					
No	26	78.8	55	69.6	0.323
Yes	7	21.2	29	36.7	
Diabetes insipidus					
No	31	93.9	69	87.3	0.303
Yes	2	6.1	10	12.7	

*p*-value (bold if significant)

*GHD* Growth hormone deficiency, *FLI* fatty liver index, *SE* standard error, *SD* standard deviation, *NA* not available

* Including five patients primary adrenal insufficiency due to adrenalectomy

### FLI as a measure of NAFLD

In the entire study population FLI correlated with female sex (r = −0.313; *p* = 0.001), Hb1Ac (r = 0.328; *p* = 0.001), fasting glucose (r = 0.404; *p* < 0.001), HDL (r = −0.425; *p* < 0.001), BMI (r = 0.852; *p* < 0.001), GGT (r = 0.499; *p* > 0.001), triglycerides (r = 0.456; *p* < 0.001), systolic (r = 0.332; *p* < 0.001) and diastolic BP (r = 0.415; *p* < 0.001), ASAT (r = 0.432; *p* > 0.001) and ALAT (r = 0.357; *p* < 0.001) but not with the ASAT/ALAT-ratio. There was no significant difference in FLI scores between patients with and without overt hypogonadism. Same was true for those with untreated GHD in comparison to those without GHD or those that were on adequate replacement therapy. There was also no significant correlation with IGF-1 levels (*p* = 0.869) in univariate analysis. FLI did not differ between patients with and without adrenal insufficiency but there was a trend for a positive correlation of average daily hydrocortisone intake (r = 0.253, *p* = 0.086).

To assess independent predictors of FLI, a multivariate regression model using backward stepwise removal was used, entering FLI as a dependent variable and age, sex, diagnosis (CD vs. NFPA), untreated hypogonadism (primary + secondary), adrenal insufficiency, average daily intake of hydrocortisone replacement, untreated GHD, IGF-1 and hypothyroidism.

In this model, the only independent predictors were sex (β = −22.535; *p* = 0.016) and average daily hydrocortisone intake (β = 1.804, *p* = 0.042). Even when BMI and waist circumference, which are part of the FLI calculation, were included in the model, average daily hydrocortisone intake remained an independent predictor of FLI scores (β = 1.124; *p* = 0.017; Table 2).

**Table 2:** Predictors of the fatty liver index

Model	β	SE	*p* value	Confidence interval (CI; 95.0 %)	
				Lowest	Highest
Model 1*					
Female sex	−22.535	8.971	0.016	−39.298	−2.006
HC dosage	1.804	0.859	0.042	0.072	3.536
Model 2**					
HC dosage	1.124	0.451	0.017	0.212	2.036
BMI	4.087	0.526	< 0.001	3.023	5.151
Waist	0.296	0.132	0.031	0.028	0.563

*β*, Partial regression coefficient; *HC*, hydrocortisone; *SE*, standard error of partial regression coefficient

* Included age, sex, diagnosis, untreated GHD, adrenal insufficiency, untreated hypogonadism, secondary hypothyroidism

** + BMI + Waist circumference

**Fig. 1:**
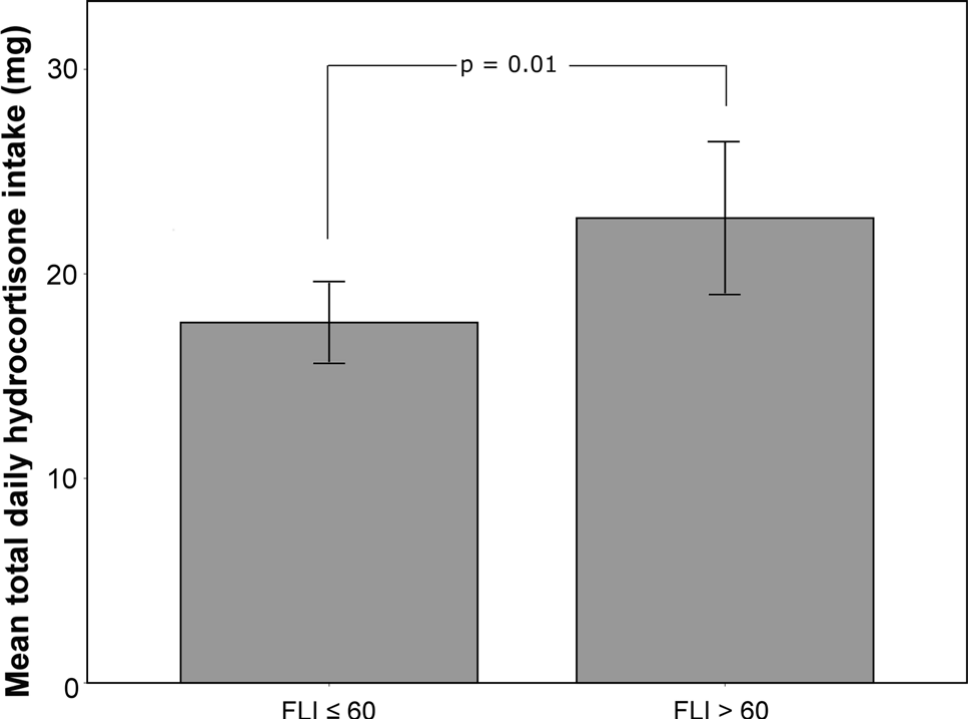
Average daily hydrocortisone intake and FLI > 60

In line, those with a FLI > 60 were also taking in average a significantly higher daily HC-dosage than those with a score < 60 (21.05 mg ± 5.9 vs. 17.9 mg ± 4.4; *p* = 0.01; Fig. 1). This finding was independent of age, sex and pituitary function (Table 3), but did however not survive if BMI and waist circumference were included in the model (data not shown).

**Table 3:** Predictors for an FLI > 60

Model with FLI (used as a dichotomic variable; FLI ≥ 60 and FLI < 60)*					
Model	β	SE	*p* value	Confidence interval (CI; 95.0 %)	
Sex (male)	2.758	0.845	0.027	0.013	00.768
HC dosage	0.201	0.082	0.02	1.041	10.587

*β*, Partial regression coefficient; *SE*, standard error of partial regression coefficient; *HC*, hydrocortisone

* Including age, sex, diagnosis, hypogonadism, secondary hypothyroidism, adrenal insufficiency, untreated GHD

### Predictors for cardiovascular risk factors

We determined which factors might predict differences in other cardio-metabolic variables such as total cholesterol levels, HDL, LDL, systolic and diastolic BP, fasting glucose and HbA1c levels in a stepwise linear regression analysis (Table 4). We performed the analysis once for the whole study population and once only for those who were not already receiving medical treatment for arterial hypertension, dyslipidemia or type 2 diabetes, respectively.

**Table 4:** Predictors of cardiovascular risk factors

Dependent variable*	All patients				Not pretreated**			
	Independent variable	β	SE	*p* value	Independent variable	β	SE	*p* value
HDL	FLI	−0.262	0.118	0.032	FLI	−0.356	0.134	0.013
					GHD	18.567	8.435	0.037
Systolic blood pressure (mmHg)	Age	0.851	0.180	<0.001	Age	0.426	0.152	0.001
					Sex	−13.622	4.488	0.012
Diastolic blood pressure (mmHg)	Sex	−10.184	2.723	0.001	Sex	−7.852	2.731	0.010
HbA1c (%)	FLI	0.008	0.002	0.001	FLI	0.235	0.064	0.001
	Age	0.014	0.005	0.006				
	Sex	0.348	0.156	0.031				
Fasting glucose (mg/dl)	FLI	0.230	0.068	0.002	FLI	0.235	0.064	0.001

*FLI* Fatty liver index

* The predictive variables were age, sex, diagnosis, FLI, hypogonadism, HC dosage and untreated GHD and IGF-1

** Patients excluded taking anti-hyperglycemic drugs (for HbA1c, fasting glucose), antihypertensive medication (for systolic, diastolic BP), anti-hyperlipidemic drugs (for total cholesterol, HDL, LDL), respectively

Every regression model included age, sex, diagnosis (CD vs. NFPA), untreated hypogonadism (primary + secondary), average dosage of hydrocortisone replacement, untreated GH deficiency, IGF-1 and FLI scores as independent variables.

FLI (*p* = 0.032) was the best single predictor for HDL-cholesterol levels. If those were excluded from the analysis that had already received anti-hyperlipidemic treatment, only FLI (*p* = 0.013) and GHD remained significant (*p* = 0.037). Age was the only independent predictor for systolic BP (*p* < 0.001), while age (*p* = 0.001) and sex (*p* = 0.012) were predictive for systolic BP in those who did not already receive antihypertensive medication. Female sex was the only independent negative predictor of diastolic BP and remained significant after excluding those with anti-hypertensive treatment (*p* = 0.001). HbA1c was independently predicted by FLI (*p* = 0.001), age (*p* = 0.006) and male sex (*p* = 0.031). FLI remained the only significant predictor if those with anti-hyperglycemic treatment were excluded from the analysis (*p* = 0.001). FLI was also the only independent predictor for fasting glucose levels for those with (*p* = 0.001) and without anti-diabetic treatment (*p* = 0.002; Table 4).

If FLI was substituted by the separate variables used for its equitation (BMI, waist circumference, triglycerides and GGT), GGT was the best determinant for HbA1c levels in the whole study cohort (*p* = 0.016) and after excluding those with pretreated diabetes mellitus type 2 (*p* = 0.012). GGT was also the best predictor for fasting glucose levels (*p* < 0.001; Table 5).

**Table 5:** Individual analysis of the components of the fatty liver index

	β	SE	*p* value
*Model for Hba1c*			
Model 1*			
GGT	0.400	0.155	0.016
Model 2**			
GGT	0.342	0.124	0.012
*Model for fasting glucose*			
Model 1*			
GGT	18.988	4.297	<0.001
Model 2**			
GGT	18.833	3.237	<0.001

The predictive variables were age, sex, diagnosis, overt hypogonadism, HC dosage and untreated GHD, triglycerides, BMI, GGT, waist circumference

* All patients

** Only patients without anti-diabetic drugs

## Discussion

In the present study we could show that HC dosage was an independent predictor of FLI as a marker of NAFLD. This association also remained significant after accounting for waist circumference and BMI. HC intake was also significantly higher in those with an FLI score >60 which has been shown to have a high predictive value for NAFLD in histological analysis [24]. This indicates that an increased hepatic exposure with hydrocortisone does not only impact hepatic lipid accumulation via an increase in visceral adiposity, which is a well-established consequence of hypercortisolism [25] and may even persist after achievement of normocortisolism [10], but also by direct effects. The regression analysis indicates that every mg of HC results in average in an independent increase of 1.1 units in the FLI. Though this at first glance seems to be only a moderate increase, it has also to be kept in mind that it has been shown in the general population that an increase of the FLI by only one unit may result in an increased chance of developing NAFLD by 5.8 %. It is known that GC treatment may result in liver fat accumulation [26], however, this has rather been reported in the context the administration of supraphysiological dosage due to its anti-inflammatory effect in inflammatory diseases [27]. Although there is also evidence for general hypothalamo-pituitary-adrenal (HPA) overactivity in NAFLD patients [28], data supporting a more tissue specific role of GCs in the pathogenesis of NAFLD are even more compelling.

These data show that direct cortisol exposure to liver is a key mediator of liver fat metabolism and NAFLD development [26]. The glucocorticoid receptor (GR) is a critical regulator of liver glucose and lipid homeostasis [29], mediating effects on hepatic lipid metabolism by directly promoting liver fat accumulation via an increase in lipoproteins secretion and up-regulation of enzymes involved in fatty acid synthesis [30] and inhibition in liver-specific fatty acid oxidation [31]. In line, liver-specific knockdown of the GR expression ameliorates steatosis severity [32].

A critical role seems to be played by the local metabolism of glucocorticoids, in particular conversion of cortisone to cortisol by the 11β-hydroxysteroid dehydrogenase type 1 (11β-HSD1)-enzyme that is primarily found in visceral adipose and liver tissue [33]. Hepatic [34] as well as visceral 11β-HSD1 [35] have both been shown to play a significant role in the etiology of the metabolic syndrome. This enzyme is in particular overexpressed in visceral fat of obese subjects resulting in cortisol-overexposure of the liver by the splanchnic venous system [36]. This is of certain importance as already in metabolically healthy individuals, splanchnic 11β-HSD1 reactivation of cortisone alone is expected to account for approximately one fourth of circulating cortisol levels [33].

Correspondingly, adipose tissue specific knock-out of 11β-HSD1 in animal models protects from the development of a metabolic syndrome-like phenotype despite systemic corticosteroid excess [35]. In turn, mice overexpressing 11β-HSD1 in the fat tissue show unaltered systemic GC levels while GC levels are increased in portal vein and as a consequence, these mice are developing impaired hepatic lipid clearance and steatosis [34]. On the one hand selective overexpression of 11β-HSD1 results in the development of all features of the metabolic syndrome expect obesity [34], on the other hand, selective knock-out of hepatic 11β-HSD1 is not sufficient to prevent features of the MS and steatosis, when visceral 11β-HSD1 is still active and animals are exposed to high doses of GC.

These findings underline the critical role of hepatic exposure to GCs for the development of NAFLD and the metabolic syndrome and have promoted the development of selective inhibitors of 11β-HSD1 [37–40].

Again, while the effects of liver specific 11β-HSD1 inhibition on metabolic parameters have been less convincing [39], the use of an 11β-HSD1 inhibition that targets both, adipose and liver 11β-HSD1 has shown beneficial results in type 2 diabetics in addition to metformin therapy in terms of significant improvements in glycemic control and dyslipidemia [40]. The importance of local and in particular visceral glucocorticoid activation is further underscored by the fact that patients who have been treated with these drugs show significant improvement in metabolic parameters despite a compensatory increase in ACTH and subsequently unaltered systemic cortisol levels. In addition, it was further demonstrated that the improvement in triglyceride levels and hemoglobin A1c was even more promising in those with a higher BMI though there was no difference in baseline metabolic parameters in this regard, potentially due to the higher 11β-HSD1 expression as a result of increased visceral adipose tissue in these subjects.

All these findings highlight the importance of hepatic GC exposure via the portal venous system and coming back to the findings of our study, it has to be kept in mind that in contrast to individuals without adrenal insufficiency, where cortisol is directly secreted from the adrenals into the systemic circulation, oral intake of HC as hormone replacement therapy results in a higher liver exposure as it will first enter the portal venous system and therefore result in a relatively increased liver exposure.

In keeping, patients with secondary adrenal insufficiency have an increased overall mortality in particular if they take more than 20 mg per day [41] and also show an unfavorable cardiometabolic risk profile. In contrast, our data do not indicate that preceding hypercortisolism as in cured CD has a sustained impact on the development of NAFLD, making other factors more likely to contribute to the poor cardiometabolic outcome reported in the literature such as persisting visceral obesity [10].

Adams and colleagues demonstrated in a longitudinal study in a small subset of patients with hypopituitarism that NAFLD development may finally resulting in NASH, fibrosis, cirrhosis and hepatocellular carcinoma, even demanding liver transplantation due to liver failure [42]. However, a large proportion of this small subgroup investigated, was suffering from hypothalamic obesity, potentially differing in terms of NAFLD pathophysiology in comparison to NFPA patients. According to the authors, almost all patients were on HC replacement therapy, but they do not report on dosage.

In contrast to the role of HC-, GHD, respectively GHD replacement therapy did not seem to have a significant influence on FLI as a marker of NAFLD in our study cohort, neither in CD nor in NFPA patients.

Nishizawa and colleagues [14] reported on a high prevalence of NAFLD of up to 70 % in GHD patients and also a high prevalence of NASH. They also documented a significant improvement in terms of liver histology and liver enzymes in a subgroup of patients receiving GH. The high prevalence of NAFLD and NASH may be explained by a high rate of patients with craniopharyngeoma included, potentially suffering from hypothalamic damage. Though controls were matched for BMI, these patients may represent a distinct entity which is not directly comparable with NFPA patients. In this patient cohort, most patients were also suffering from adrenal insufficiency but the authors do not report on replacement regimens. In contrast Garnder et al. [13] who estimated NAFLD by means of magnetic resonance spectroscopy did not find an increased prevalence of NAFLD in comparison to matched controls. In this cohort only a minority of patients was suffering from adrenal insufficiency. We can only speculate on the discrepant findings in these studies but etiology of GHD and extent of accompanying pituitary insufficiencies and subsequent replacement regimens may play a role in this context. It has to be kept in mind that there is a close interaction between the different hormonal axes. GH for example is known to suppress 11β-HSD1 activity and therefore reducing cortisol exposure.

FLI in our cohort was an independent predictor for conventional cardiovascular risk factors such as HbA1c, fasting glucose and HDL cholesterol. These findings are in accordance with the literature [24]. HbA1c and fasting glucose were best predicted by GGT-levels if analysis of the four factors contributing to its calculation were investigated separately. This finding is of interest as GGT levels have also been suggested to be predictive for mortality also independent of the presence of NAFLD [41].

Apart from the obvious that HC replacement dosage in patients with adrenal insufficiency should be as high as necessary but as low as possible, the clinical implications of our findings might include to further investigate the effects of different HC-formulations and application routes such as modified-release HC and HC via continuous subcutaneous infusion (CSI) on NAFLD development. Modified-release HC has shown beneficial effects with regard to metabolic outcome [43–45], although it is still not completely clear if this is solely due to a reduced overall bioavailability or due to the more physiological HC release. It has been shown that patients who had switched from conventional to a dual-release regimen may benefit in terms of cardiometabolic parameters without negative effects on quality of life [43]. Studies comparing conventional HC to extended-release HC showed in particular significantly lower levels of triglycerides and BMI [43, 44], both being part of the FLI calculation while others have shown a reduction in waist circumference but not on triglycerides [45].

On the other hand if you follow our argumentation that the direct hepatic HC exposure via the portal circulation may have more detrimental effects with regard to NAFLD development, the administration of HC by CSI should result in benefits with regard to liver steatosis. The so far available pilot studies on HC administration via CSI did not investigate the effects on NAFLD, but showed no benefits with regard to glucose metabolism that is at least pathophysiologically closely related to liver steatosis as also being shown in our study by the FLI and GGT being best predictor for fasting glucose and HbA1c levels. The interpretation of the results of these studies is however hampered by the fact that in one study CSI resulted in a significant increased overall HC exposure in comparison to the oral dosage potentially counteracting any beneficial effects exerted by the different route of application and in the other study area under the curve (AUC) for cortisol exposure was only determined during the euglycaemic-hyperinsulinaemic clamp test [46]. It remains therefore speculative if a same-dose exposure to HC given parenterally would be better for liver and metabolic health than oral application.

There are some limitations that have to be taken into consideration for the interpretation of our results. The major limitation refers to the use of an index for determining NAFLD. Whilst non-invasive assessments of NAFLD as the FLI are able to provide accurate measurements of increased liver fat, it cannot replace staging of disease by liver biopsy as they have lower accuracy in quantifying steatosis. However, the index has been proven highly accurate in detecting a fatty liver [18] and most studies in western populations have shown that a FLI < 30 has an accuracy for excluding NAFLD between 80 and 87 % while a cut off of >60 has a specificity of 80 % for detecting NAFLD, though cut-off points may be different [20] in other ethnic populations [47, 48]. The FLI has initially been developed in a large epidemiological study from the general population and was tested against the diagnosis of a fatty liver by a single-operator ultrasonography (US) according to four stringent US-criteria [49] that in turn had shown to diagnose NAFLD defined by a histological confirmed fat content of >30 % with a sensitivity of 83 % and specificity of 100 % [18]. US has shown comparable rates of sensitivity in comparison to other advance imaging techniques such as 1H-MRS, although the specificity may be lower [50]. The FLI has been shown to perform equally good in men and women [47, 48], though some authors did find different cut-off points depending on sex [48].

Indirect measures of hepatic steatosis by indexes such as the FLI but also by imaging techniques fail at making the important distinction between NAFLD and NASH [20, 51]. However, due to the associated high costs, sampling error and morbidity with liver biopsies, this approach according to current consensus guidelines is only justified if clinical history, examination, laboratory results and abdominal ultrasound [52] indicate sever NAFLD or NASH.

In addition to its application for detecting NAFLD, the index has been shown to be a useful predictor of arteriosclerosis development, independent of established classical risk factors in particular in those patients with a score ≥ 60 [53], and may also be an independent predictor for the development of arterial fibrillation [54], diabetes [55], cardiovascular and all-cause mortality [21, 24]. If these results are directly transferable to patients with hypopituitarism with their unique hormonal setting demands however further evaluation.

A further limitation is the cross-sectional non-controlled design of our study. Reasons for not using GHD replacement therapy are various and may range from missing subjective or objective benefit from treatment as well as development of side effects or a general refusal of daily injections. Further prospective studies including a separate analysis of HC replacement regimes are therefore needed to evaluation the obviously complex role of GHD and HC in NAFLD among patients with pituitary dysfunction. Despite these limitations, we believe our data provide important implications for a better understanding NAFLD in general and its management of in patients with pituitary insufficiencies.

## Conclusion

In summary we could show that while HC dosage affects FLI as an estimate of NAFLD in patients with pituitary adenomas the benefit of GH replacement still needs to be determined. In contrast to reports in CD patients with active disease, NAFLD in those biochemically controlled was not significantly increased in comparison to NFAP patients. Our study also contributes to the understanding of the role of cortisol in the development of NAFLD. Further studies should assess the diagnostic accuracy in comparison to histological criteria and its prognostic implications in this special patient population.
